# A Panel of Novel Biomarkers Representing Different Disease Pathways Improves Prediction of Renal Function Decline in Type 2 Diabetes

**DOI:** 10.1371/journal.pone.0120995

**Published:** 2015-05-14

**Authors:** Michelle J. Pena, Andreas Heinzel, Georg Heinze, Alaa Alkhalaf, Stephan J. L. Bakker, Tri Q. Nguyen, Roel Goldschmeding, Henk J. G. Bilo, Paul Perco, Bernd Mayer, Dick de Zeeuw, Hiddo J. Lambers Heerspink

**Affiliations:** 1 Department of Clinical Pharmacy & Pharmacology, University of Groningen, University Medical Center Groningen, Groningen, The Netherlands; 2 emergentec biodevelopment GmbH, Vienna, Austria; 3 Center For Medical Statistics, Informatics, And Intelligent Systems, Medical University of Vienna, Vienna, Austria; 4 Diabetes Centre, Isala Clinics, Zwolle, The Netherlands; 5 Department of Gastroenterology and Hepatology, University of Groningen, University Medical Center Groningen, Groningen, The Netherlands; 6 Department of Internal Medicine, Division of Nephrology, University of Groningen, University Medical Center Groningen, Groningen, The Netherlands; 7 Department of Pathology, University Medical Center Utrecht, Utrecht, The Netherlands; Nanyang Technological University, SINGAPORE

## Abstract

**Objective:**

We aimed to identify a novel panel of biomarkers predicting renal function decline in type 2 diabetes, using biomarkers representing different disease pathways speculated to contribute to the progression of diabetic nephropathy.

**Research Design and Methods:**

A systematic data integration approach was used to select biomarkers representing different disease pathways. Twenty-eight biomarkers were measured in 82 patients seen at an outpatient diabetes center in The Netherlands. Median follow-up was 4.0 years. We compared the cross-validated explained variation (R^2^) of two models to predict eGFR decline, one including only established risk markers, the other adding a novel panel of biomarkers. Least absolute shrinkage and selection operator (LASSO) was used for model estimation. The C-index was calculated to assess improvement in prediction of accelerated eGFR decline defined as <-3.0 mL/min/1.73m^2^/year.

**Results:**

Patients’ average age was 63.5 years and baseline eGFR was 77.9 mL/min/1.73m^2^. The average rate of eGFR decline was -2.0 ± 4.7 mL/min/1.73m^2^/year. When modeled on top of established risk markers, the biomarker panel including matrix metallopeptidases, tyrosine kinase, podocin, CTGF, TNF-receptor-1, sclerostin, CCL2, YKL-40, and NT-proCNP improved the explained variability of eGFR decline (R^2^ increase from 37.7% to 54.6%; *p*=0.018) and improved prediction of accelerated eGFR decline (C-index increase from 0.835 to 0.896; *p*=0.008).

**Conclusions:**

A novel panel of biomarkers representing different pathways of renal disease progression including inflammation, fibrosis, angiogenesis, and endothelial function improved prediction of eGFR decline on top of established risk markers in type 2 diabetes. These results need to be confirmed in a large prospective cohort.

## Introduction

The growing prevalence of type 2 diabetes is a great global health problem. Type 2 diabetes is the leading cause of chronic kidney disease (CKD) in the United States and is associated with high cardiovascular risk [1, 2]. Optimizing treatment has been shown to improve life expectancy, reduce costs, and lower the risk of death in patients with type 2 diabetes [3, 4]. Despite important progress in improving therapy, many patients are still at risk for renal disease.

Early identification of patients with type 2 diabetes at risk for progressive renal function loss during the early stages of disease may lead to better patient outcomes. In clinical practice, estimated glomerular filtration rate (eGFR) and albuminuria are used to assess renal function when gold-standard measured GFR is not feasible or practical. The search for novel biomarkers that improve risk prediction models on top of established risk markers has been a priority of many researchers for many years. Various studies have assessed the performance of single biomarkers representing a single, disease-associated pathway to predict progression of renal function loss in type 2 diabetes [5, 6]. However, because type 2 diabetes is a multifactorial disease, several pathways involving pro-inflammatory, pro-fibrotic, and angiogenic processes, among others, are activated during the course of the disease [7]. Given the complexity of the multiple pathophysiological processes involved in progression of type 2 diabetes together with the intra-individual variability of biomarkers, it is questionable if a single biomarker may possess useful diagnostic and prognostic power. Alternatively, a combination of biomarkers that capture different pathways of renal damage may provide a more realistic picture of a patient’s actual pathophysiological status and hence may yield better assessment of disease prognosis performance.

Therefore, we aimed to identify a novel panel of biomarkers representing different disease pathways that are speculated to contribute to the progression of renal disease in type 2 diabetes, and to evaluate their combined predictive performance of accelerated renal function decline.

## Research Design and Methods

### Patients and methods

This observation cohort study was performed in Caucasian patients from Zwolle, The Netherlands, who participated in the PREvention of DIabetic ComplicaTIONS (PREDICTIONS) study [8]. Patients aged 35–75 with type 2 diabetes with a documented duration of ≥5 years were eligible for the PREDICTIONS study. Type 2 diabetes was defined according to World Health Organization criteria [9]. A total of 82 patients were recruited in 2007–2008 and followed for a median of 4.0 [1^st^, 3^rd^ quartile 3.7 to 4.4] years. Follow-up information on urinary albumin:creatinine ratio (UACR), serum creatinine, cholesterol, and glycated hemoglobin (HbA_1c_) was obtained from electronic patient files from visits to the outpatient diabetes clinic during their annual visit to the diabetes specialist.

### Ethics Statement

The PREDICTIONS study was approved by the ethical review boards of the medical ethics committees of the Isala Clinics in Zwolle and of the University Medical Center in Groningen, The Netherlands and was conducted in accordance with the guidelines of the Declaration of Helsinki. All patients gave written, informed consent.

### Selection of biomarkers, sample collection, preparation, and measurement

Twenty-eight biomarkers were selected for testing using three distinct approaches, namely a literature review [10], identification of molecular processes and pathways [7], and ranking of consolidated Omics signatures [11]. A complete list of biomarkers is presented in Table 1, and the biomarker selection procedure is described in S1 Appendix.

**Table 1:** Concentrations of biomarkers* and univariate and multivariable associations of single biomarkers with eGFR decline.

		Concentrations	Univariate association			Multivariable association^†^		
Pathway	Biomarker	Median [1^st^, 3^rd^ quartile]	β	95% CI	*p-*value	β	95% CI	*p-*value
*Inflammation*								
Monocyte chemoattractant protein-1 (CCL2) (pg/mL)		316.2 [258.3, 386.4]	-1.1	-3.6, 1.5	0.41	0.1	-2.0, 2.3	0.89
Tumor necrosis factor receptor-1 (TNFR1) (ng/mL)		3.8 [3.1, 6.7]	-3.2	-5.2, -1.2	**<0.01**	-2.1	-4.0, -0.3	**0.03**
Tumor necrosis factor receptor-2 (TNFR2) (pg/mL)		317.6 [248.3, 475.0]	-2.4	-3.8, -0.9	**<0.01**	-2.2	-4.6, 0.3	0.08
Chitinase 3-like 1 (YKL-40) (ng/mL)		36.5 [21.1, 87.1]	-1.1	-1.9, -0.2	**0.01**	-0.5	-1.3, 0.3	0.20
Chemokine (C-X-C motif) 1 (CXCL1) (pg/mL)		80.0 [71.1, 94.7]	1.6	-1.7, 4.8	0.33	-0.2	-3.0, 2.6	0.88
Chemokine (C-X-C motif) 10 (CXCL10) (pg/mL)		80.6 [58.1, 121.7]	-0.5	-1.7, 0.6	0.35	-0.5	-1.4, 0.5	0.35
*Fibrosis*								
Connective tissue growth factor (CTGF) (nmol/L)		1.0 [0.8, 1.5]	-3.4	-7.8, 0.3	0.07	-3.8	-7.7, 0.1	0.05
Matrix metallopeptidase 1 (MMP1) (pg/mL)		767.4 [470.0, 1315.5]	-0.4	-1.4, 0.7	0.49	0.2	-0.8, 1.2	0.72
Matrix metallopeptidase 2 (MMP2) (ng/mL)		38.3 [36.1, 40.0]	9.6	0.4, 18.7	**0.04**	5.6	-1.9, 13.1	0.14
Matrix metallopeptidase 7 (MMP7) (ng/mL)		1.6 [0.6, 3.0]	-1.4	-2.1, -0.7	**<0.01**	-0.8	-1.5, -0.04	**0.04**
Matrix metallopeptidase 8 (MMP8) (ng/mL)		2.4 [1.5, 4.8]	0.1	-0.8, 0.7	0.88	0.3	-0.3, 0.9	0.34
Matrix metallopeptidase 13 (MMP13) (pg/mL)		120.0 [108.5, 142.4]	-1.2	-3.0, 0.7	0.23	-0.7	-2.4, 1.0	0.40
Podocin (NPHS2) (ng/mL)		0.9 [0.3, 1.2]	-2.9	-5.0, -0.7	**0.01**	-0.9	-3.0, 1.3	0.44
Leptin (LEP) (ng/mL)		15.9 [10.1, 33.3]	0.2	-0.6, 0.9	0.66	-0.3	-1.1, 0.6	0.54
*Angiogenesis*								
Endostatin (Frag.COL18A1) (pmol/L)		7.6 [6.3, 9.7]	-2.6	-5.0, -0.3	**0.03**	-1.3	-4.4, 1.7	0.39
Tyrosine kinase (TEK) (pg/mL)		666.9 [315.7, 1275.8]	-0.6	-1.6,0.3	0.18	-0.9	-1.7, -0.1	**0.03**
Vascular endothelial growth factor-A (VEGF-A) (pg/mL)		66.7 [30.6, 155.9]	-0.5	-1.1, 0.2	0.13	-0.2	-0.8, 0.4	0.49
Hepatocyte growth factor (HGF) (pg/mL)		65.9 [35.0, 120.3]	-0.7	-1.5, 0.2	0.11	-0.2	-0.9, 0.5	0.61
*Endothelial Dysfunction*								
Amino terminal pro C-type natriuretic peptide (NT-proCNP)(pmol/L)		2.9 [2.3, 4.3]	-1.3	-2.9, 0.3	0.12	-0.7	-2.4, 0.9	0.38
*Mineral metabolism*								
Fibroblast growth factor 23 (FGF23) (pmol/L)		4.0 [2.6, 5.5]	-0.8	-2.1, 0.5	0.24	-0.2	-1.3, 1.0	0.78
Sclerostin (SOST) (pmol/L)		42.7 [33.4, 52.2]	-0.1	-2.4, 2.3	0.96	0.3	-1.6, 2.3	0.75
*Lipid metabolism*								
Zinc-binding alpha-2-glycoprotein 1 (AZGP1) (ng/mL)		13.4 [9.0, 20.2]	-1.1	-2.5, 0.4	0.14	-0.6	-1.8, 0.7	0.37
*Glomerular damage*								
Growth hormone 1 (GH1) (pg/mL)		330.8 [53.4, 994.2]	-0.2	-0.7, 0.3	0.38	-0.3	-0.7, 0.1	0.14

*Concentrations of nephrin (NPHS1), neuropilin-1 (NRP1), interleukin-1 alpha (IL1A), interleukin-1 beta (IL1B), and epidermal growth factor (EGF) were missing in 10% of observations or undetectable in >25% of observations, and these biomarkers were therefore not used in analysis.

^†^Adjusted for established risk markers: baseline UACR, current vs. never smoker, sex, systolic blood pressure, use of oral diabetic medication, diastolic blood pressure, and baseline eGFR.

Fasting serum and plasma samples were stored at -80°C. All samples were stored for 4–5 years and did not undergo any freeze-thaw cycles. Biomarkers were assayed on baseline samples by enzyme-linked immunosorbent assay (ELISA) or multiplex assay by Biomarker Design Forschungs GmbH (BDF), in Vienna, Austria, except for connective tissue growth factor (CTGF). CTGF was measured using specific antibodies (FibroGen Inc., San Francisco, USA) directed against distinct epitopes in the amino-terminal fragment of CTGF, as described previously [12]. All assays were used according to manufacturer’s instructions. A complete list of assays, and information on stability and determination of limits of detection are available in S2 Appendix. All biomarker analyses were performed blinded, and the results were then reported back to the study center for analysis.

### Statistical analysis

Analyses were performed with SAS software (version 9.2; SAS Institute, Cary, NC) and R version 3.0.2 [13] using the packages mice and glmnet [14, 15]. Data are presented as mean (standard deviation) or median [1^st^, 3^rd^ quartile] for skewed variables. Graphical techniques were used to detect outliers. The natural logarithm of UACR and the binary logarithm of all biomarkers were used to normalize their distributions. Log transformed variables were used in all regression analysis. Values below the detection limit were set to the detection limit. Variables with missing values were multiply imputed using chained equations [16]. Five of the twenty-eight biomarkers had values with >10% missing or >25% below the detection limit were not used in analysis. Details on our implementation of multiple imputation can be found in S3 Appendix. All *p*-values were two-tailed, and values < 0.05 were considered statistically significant.

The outcome of interest was eGFR decline, defined as the within-patient annual eGFR slope. EGFR decline was calculated using a minimum of 3 serum creatinine measurements during follow-up by fitting a straight line through the eGFR values using linear regression. The eGFR value at each time-point was estimated using the 4-variable Modification of Diet in Renal Disease (MDRD) Study Equation [17].

Statistical modeling consisted of several steps. First, established risk markers were selected as best predictors of eGFR decline using least absolute shrinkage and selection operator (LASSO) selection [18]. The LASSO is advantageous for small samples sizes because it places restrictions on the absolute sizes of the regression coefficients in the model while optimally selecting the subset of variables that best predicts the outcome. This restriction also controls for multicollinearity. LASSO involves the estimation of a tuning parameter controlling the amount of restriction, which was optimized by minimizing the leave-one-out cross-validated mean squared error of prediction. The established risk markers listed in Table 2 were considered as potential predictors of eGFR decline. All established risk markers were first included in a multivariable model using LASSO regression. The best predictors of eGFR decline were then identified from the multivariable model and are reported in the results section. Second, univariate linear models were fit for each of the novel biomarkers to assess a single biomarker association with eGFR decline. Third, multivariable models were then fit by linear regression with single novel biomarkers adjusting for the selected established risk markers. Fourth, a multivariable model including the selected established risk markers and all biomarkers was fit using the LASSO selection in order to find the best subset of predictors.

**Table 2:** Baseline characteristics in patients with type 2 diabetes (n = 82).

Risk marker	Baseline values
Age (years)	63.5 ± 9.4
Male Gender (%)	44 (53.7)
Current smoker (%)	8 (9.6)
Body mass index (kg/m^2^)	32.4 ± 6.3
Systolic blood pressure (mmHg)	135.2 ± 16.3
Diastolic blood pressure (mmHg)	72.7 ± 10.5
Duration of diabetes (years)	15.7 ± 7.3
*Baseline laboratory measurements*	
UACR (mg/mmol)	1.2 [0.5, 57.7]
Serum creatinine (μmol/L)	88.4 ± 33.5
eGFR (mL/min/1.73m^2^)	77.9 ± 22.6
HDL Cholesterol (mmol/L)	1.3 ± 0.4
LDL Cholesterol(mmol/L)	2.0 ± 0.6
HbA_1c_ (%)	7.7 ± 1.3
*Medication use*	
RAAS* (%)	27 (42.9)
Insulin* (%)	58 (92.1)
Oral diabetic medication* (%)	35 (55.6)

Data are reported as mean ± standard deviation or number (percent) or median [1^st^, 3^rd^ quartile].

*Data available for n = 63.

Bootstrap validation was performed to determine the validity of the model to assess the ability of the biomarker panels to predict renal function decline. The bootstrap (N = 1000) was used to evaluate selection probabilities of each biomarker, and to construct 95% confidence intervals and two-sided *p-*values for the regression coefficients by the percentile method. A global *p-*value testing the global null hypothesis of no added value of the biomarkers was constructed by counting the number of bootstrap resamples in which the multivariable biomarker model led to a smaller cross-validated mean squared error (MSE) than a model based on the established risk markers alone. In a simple bootstrap validation, LASSO models were fit to the 1000 bootstrap resamples, each time optimizing the cross-validated MSE as described above. These models were then applied to the original data without modification. The resulting MSE was calculated by averaging the squared average difference between the original outcome and the predicted outcome for each patient. This was done for models only considering the established risk markers, and for models considering clinical and biomarker predictors. From the MSEs, R^2^ measures were finally derived in order to determine whether the biomarkers significantly improved prediction.

The added value of the biomarker panel was also evaluated using the discriminative index (C-index) by dichotomizing the observed outcome variables into accelerated or non-accelerated renal function decline (eGFR decline <-3 or >-3 mL/min/1.73m^2^/year, respectively) and comparing this with predicted probabilities of eGFR decline (see S3 Appendix). The C-index was also calculated using the simple bootstrap validation scheme, and the differences in the C-index between a model of only established risk markers and a model of established risk markers plus biomarkers were assessed. The threshold of -3 mL/min/1.73m^2^ was based on prior studies and its concurrence with the high quartile of eGFR decline [19, 20].

## Results

### Baseline characteristics and association with eGFR decline

Baseline characteristics are presented in Table 2. The average age of the cohort was 63.5 (SD 9.4) years and 53.7% were male. Type 2 diabetes was well established in the study population with average diabetes duration of 15.7 (SD 7.3) years. Renal function was relatively preserved in the cohort with an average eGFR of 77.9 (SD 22.6) mL/min/1.73m^2^ at baseline. Median UACR was 10.6 [1^st^, 3^rd^ quartile: 4.42, 510.1] mg/g. The average rate of eGFR decline over the median of 4.0 [1^st^, 3^rd^ quartile: 3.7, 4.4] years of follow-up was -2.1 (SD 4.5) mL/min/1.73m^2^/year.

The following best predictors of eGFR decline were selected from the LASSO selection: baseline UACR, current vs. never smoker, sex, systolic and diastolic blood pressure, use of oral diabetic medication, and baseline eGFR (S1 Fig).

### Biomarker concentrations and associations with eGFR decline

Baseline biomarker concentrations and univariate associations of the single biomarkers with eGFR decline are reported in Table 1. Higher concentrations of the individual biomarkers matrix metallopeptidases 2 (MMP2) (*p* = 0.04), matrix metallopeptidases 7 (MMP7) (*p* < 0.01), chitinase 3-like 1 (YKL-40) (*p* = 0.01), tumor necrosis factor receptor-1 (TNFR1) (*p* < 0.01), podocin (NPHS2) (*p* = 0.01), and endostatin (frag.COL18A1) (*p* = 0.03) were significantly associated with eGFR decline. When single biomarkers were modeled adjusting for established risk markers, MMP7, tyrosine kinase (TEK), and TNFR1 were independently associated with eGFR decline (Table 1). For every two-fold increase in the log concentration of MMP7, TEK, or TNFR1, a corresponding decrease of eGFR of 0.77 (*p =* 0.04), 0.90 (*p =* 0.02), and -2.1 (*p =* 0.03) mL/min/1.73m^2^/year, respectively, was observed.

When these three biomarkers were modeled on top of the established risk markers, they did not improve the explained variability (R^2^) of eGFR decline (35.7% compared to 37.7% of the reference model; *p =* 0.988). The three biomarkers also did not increase the C-index for prediction of accelerated renal function decline (0.860 compared to 0.835 of the reference model; *p =* 0.262).

### Selection of optimal combination of established risk markers and biomarkers

**Fig 1:**
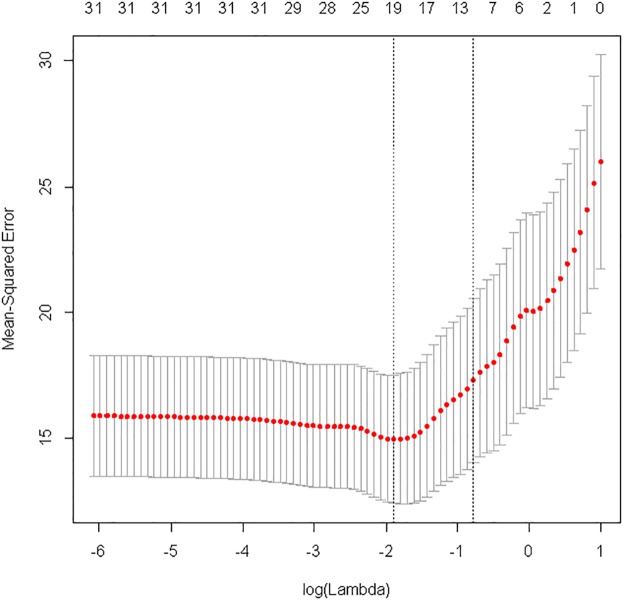
LASSO selection of optimal model of established risk markers and biomarkers: cross validated mean squared error (Y-axis; red bullets; MSE) vs. amount of restriction (X-axis; log(Lambda)). Vertical bars refer to standard errors across the 82 cross-validations. The best cross-validated MSE was obtained after inclusion of 19 variables (step 31), which included baseline UACR, MMP7, current vs. never smoker, sex, TEK, MMP2, systolic blood pressure, baseline eGFR, TNFR1, NPHS2, CTGF, use of oral diabetic medication, YKL-40, MMP1, MMP13, MMP8, SOST, CCL2, and NT-proCNP.

**Fig 2:**
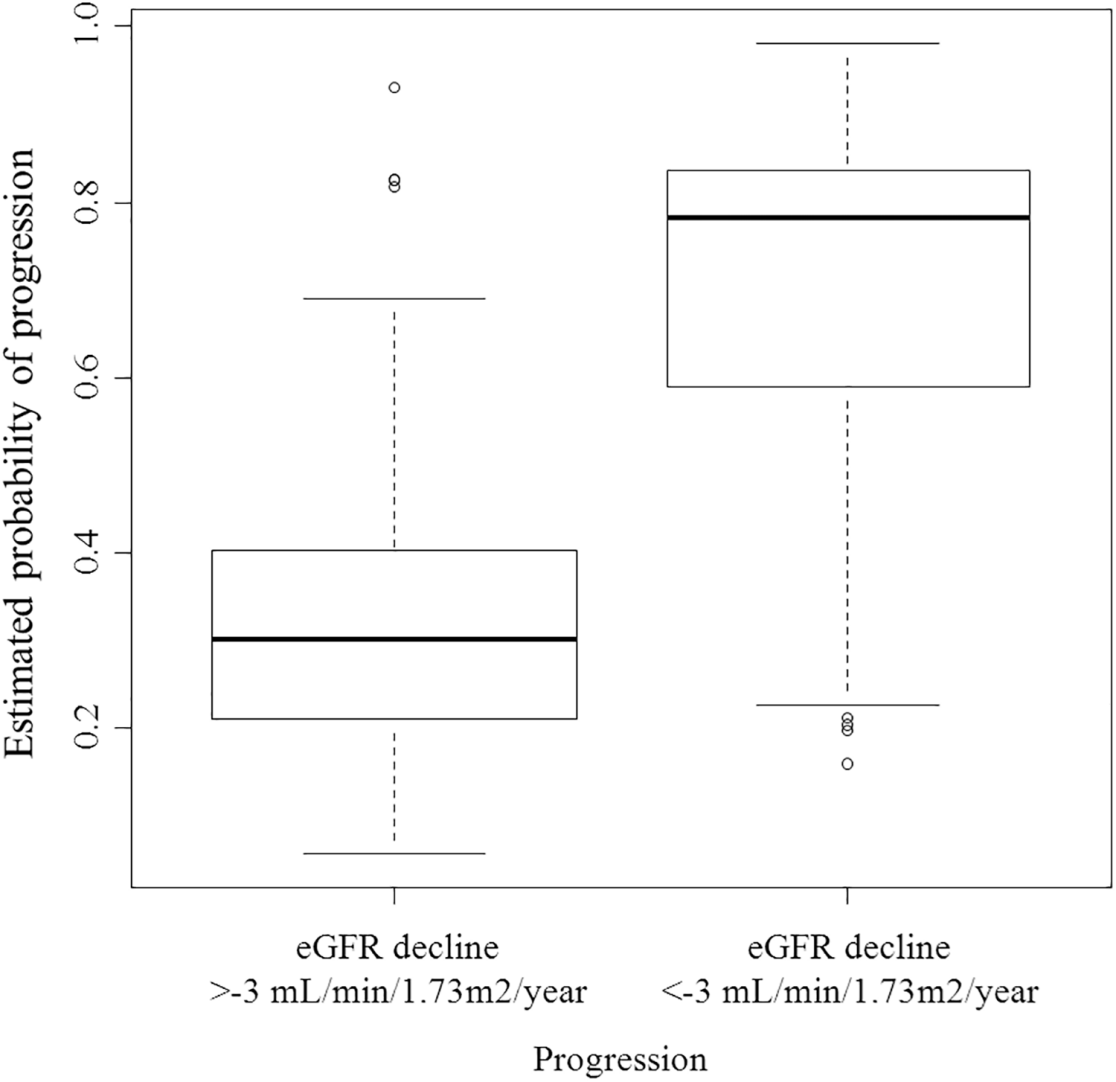
Predicted probability of accelerated renal function decline (eGFR decline <-3 or >-3 mL/min/1.73m^2^/year) in patients with type 2 diabetes.

**Fig 3:**
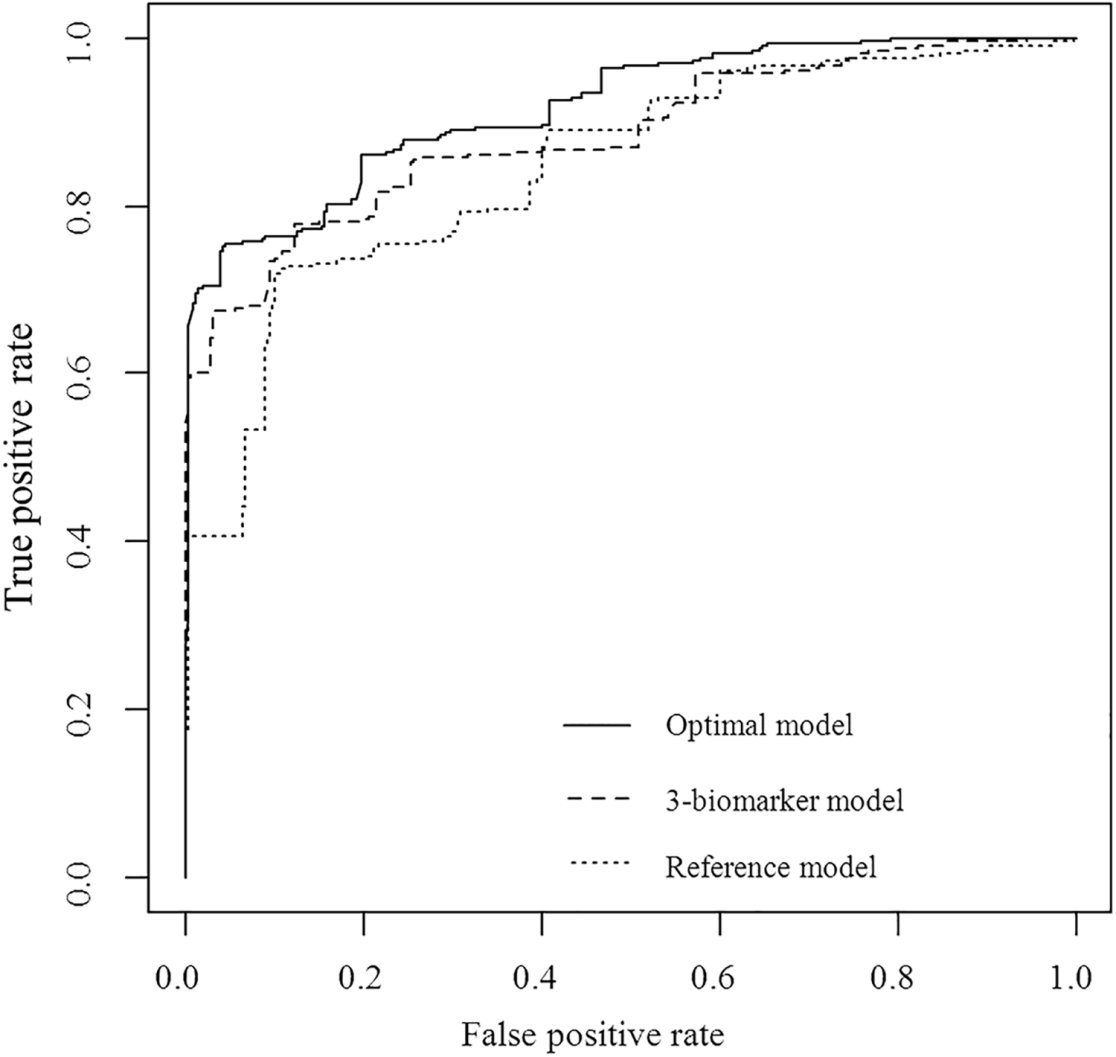
C-index for prediction of accelerated renal function decline (eGFR decline <-3 or >-3 mL/min/1.73m^2^/year) for a) established risk markers (reference model: baseline UACR, current vs. never smoker, sex, systolic and diastolic blood pressure, use of oral diabetic medication, and baseline eGFR) (C-index = 0.835), b) 3-biomarker model (MMP7, TEK, and TNFR1 on top of reference model) (C-index = 0.835; p = 0.262 compared to reference model), and c) Optimal model (baseline UACR, MMP7, current vs. never smoker, sex, TEK, MMP2, systolic blood pressure, baseline eGFR, TNFR1, NPHS2, CTGF, use of oral diabetic medication, YKL-40, MMP1, MMP13, MMP8, SOST, CCL2, and NT-proCNP) (C-index = 0.896; *p =* 0.008 compared to reference model).

Although most individual biomarkers were not found to be independently associated with eGFR decline, we hypothesized that the combination of biomarkers representing different disease pathways may improve prediction of eGFR decline. In a multivariable LASSO selection, the optimal model for prediction of eGFR decline was achieved after inclusion of 19 variables (Fig 1). The model included a subset of 13 novel biomarkers representing fibrosis, angiogenesis, inflammation, mineral metabolism, and endothelial function that, when added to the established risk markers, more accurately predicted the rate of eGFR decline (Table 3). The explained variability of the model (R^2^) markedly increased from 37.7% to 54.6% (*p =* 0.018) and predicted a higher probability of accelerated renal function decline (Fig 2). There was also a significant improvement in the C-index of the optimal model for prediction of accelerated renal function decline (0.896 compared to 0.835 of the reference model; *p =* 0.008) (Fig 3).

**Table 3:** Optimal model of established risk markers and biomarkers, results from LASSO selection and bootstrap resampling (N = 1000).

Variable	mean β	95% CI*	*p*	Selection probability^†^
Baseline UACR	-0.509	-0.834, -0.159	0.002	0.999
Systolic blood pressure	0.049	0.010, 0.085	0.012	0.994
MMP2	7.382	0.010, 0.085	0.018	0.993
TEK	-0.793	-1.416, -0.139	0.018	0.993
Baseline eGFR	-0.072	-0.130, -0.014	0.026	0.987
CTGF	-5.911	-10.358, -0.913	0.026	0.987
MMP7	-0.540	-1.191, 0.0	0.078	0.966
Current vs. never smoker	-1.593	-3.905, 0.0	0.144	0.943
MMP8	0.472	0.0, 1.036	0.134	0.935
NPHS2	-1.509	-3.667, 0.0	0.206	0.908
MMP1	0.392	-0.081, 1.051	0.298	0.897
TNFR1	-1.618	-4.037, 0.0	0.228	0.889
SOST	0.983	-0.014, 2.556	0.278	0.888
Oral diabetic medication	-1.060	-2.673, 0.0	0.274	0.884
MMP13	-0.363	-1.835, 1.020	0.798	0.820
Sex	0.792	-0.905, 2.814	0.592	0.785
CCL2	0.461	-1.228, 2.672	0.854	0.781
YKL-40	-0.405	-1.358, 0.019	0.518	0.771
NT-proCNP	0.756	-0.002, 2.452	0.568	0.742

*95% confidence interval, estimated from the 2.5^th^ and 97.5^th^ percentiles of the bootstrap distribution.

^†^The relative frequency of the marker being included in the model across 1000 bootstrap resamples.

To investigate the importance of each of the predictors in the optimal model, we omitted, one by one, variables from the full model. If a variable was omitted from the model, the other predictor variables could be selected instead. Only the omission of UACR or systolic blood pressure resulted in relevant inclusions of other novel biomarkers (S1 Table).

## Discussion

In this study, we established that a combination of different biomarkers representing different pathways that are speculated to be involved in the progression of renal disease improves prediction of eGFR decline. Although some biomarkers were not independently associated with eGFR decline, when combined into a multi-biomarker model, the combination of biomarkers improved renal risk stratification, suggesting that these biomarkers may possess synergistic effects in predicting renal function loss.

Diabetic kidney disease is characterized by the functional impairment and structural remodeling of the kidney and is linked to the changes in the kidney. Diabetic nephropathy is well characterized by glomerular hypertrophy and hyperfiltration, inflammation of glomeruli and tubuliointerstitial regions, and reduction of cell number by apoptosis and accumulation of extracellular matrix (ECM). Each of the biomarkers selected in the optimal model has been associated with one of these pathophysiological processes involved in diabetic nephropathy.

First, chronic inflammation has long been identified in the pathogenesis of type 2 diabetes and progression of diabetic nephropathy, and inflammation is well represented by the biomarkers included in the optimal model. Tumor necrosis factor alpha is a key mediator of inflammation and plays a role in apoptosis. It mediates its signal via two distinct receptors, TNFR1 and TNFR2. Circulating forms of both TNF receptors were recently shown to predict ESRD in type 2 diabetes[6]. Monocyte chemoattractant protein-1 (CCL2), another marker of inflammation, is a potent C-C chemokine for monocyte/macrophages and T cells. Increased amounts of CCL2 have been detected in renal biopsies and urine from patients with diabetic nephropathy [21], and CCL2 has been shown to be a marker of late stage diabetic nephropathy [22]. Currently there are a couple of clinical trials ongoing that target CCL2 receptor as a means to delay progression of diabetic nephropathy (www.clinicaltrials.gov identifier NCT01712061, NCT01752985). Results of these studies will provide more insight whether CCL2 is a causal factor or consequence of renal function loss. Additionally, YKL-40, a proinflammatory marker, has been identified as an independent factor associated with albuminuria in early stage nephropathy in type 2 diabetes and might have a useful role as a noninvasive marker for the early diabetic nephropathy detection [5, 23]. High YKL-40 levels have been shown to predict mortality in patients with type 2 diabetes [24]. Future mechanistic studies exploring the interplay between different inflammatory markers will help determine which markers are causal factors or consequences in the progression of diabetic kidney disease.

Second, the optimal model included several biomarkers linked to pro-fibrotic processes. Fibrosis, resulting from expansion and change in composition of ECM in the kidney, is a well-known pathologic feature of diabetic complications. Altered expression of matrix metalloproteinases (MMPs) have been implicated in the progression of diabetic nephropathy by affecting the breakdown and turnover of ECM. In mice, the overexpression of MMP-9 has been shown to induce podocyte dedifferentiation, interrupt podocyte cell integrity, and promote podocyte monolayer permeability to albumin and extracellular matrix protein synthesis [25]. In humans, serum MMP7 has been shown to be increased in diabetic renal disease and diabetic diastolic dysfunction [26]. In support of this, our study showed that higher concentrations of MMP7 were independently associated with eGFR decline. CTGF is another well investigated pro-fibrotic biomarker that was included in the optimal model. CTGF, which is upregulated in diabetic nephropathy and contributes to extracellular matrix accumulation, has been associated with both early and late stage diabetic nephropathy [12, 22]. Down-regulation of CTGF and vascular endothelial growth factor-A (VEGF-A) in diabetic nephropathy is speculated to be a result of podocyte loss [27]. Our data, in conjunction with data from literature, support the importance of fibrotic pathways in the initiation and progression of diabetic kidney disease.

Third, we included a marker representing angiogenesis. Angiogenesis is the formation of new blood vessels from pre-existing vasculature. Neovascularization has been implicated in the genesis of diverse diabetic complications such as retinopathy, impaired wound healing, neuropathy, and diabetic nephropathy. In both physiological and pathological angiogenesis, tyrosine kinase (TEK) plays a key role. TEK is principally expressed in endothelial cells and inhibits vascular permeability and tightens preexisting vessels [28]. Additionally, TEK plays a critical role in the angiogenesis of endothelial cells via binding to angiopoietin [29].

Finally the model included a marker representing endothelial function. Endothelial dysfunction is considered an initial step of the atherosclerotic process because diabetes substantially impairs vasodilating properties of the endothelium which leads to impaired vasodilation and ultimately endothelial dysfunction [30]. C-type natriuretic peptide (CNP), a member of the natriuretic peptide family, is produced in vascular endothelium. Our study implies that natriuretic (NT)-proCNP, the N-terminal fragment of the C-type natriuretic peptide precursor, contributes to prediction of eGFR decline. NT-proCNP has been shown to be associated with arterial stiffness, endothelial dysfunction, and early atherosclerosis [31], however the link of NT-proCNP to type 2 diabetes and nephropathy is still under investigation.

In our study, most biomarkers were not able to individually predict eGFR decline after adjustment for established risk markers, and the model of 3 biomarkers did not statistically improve prediction. Rather, the optimal model of 13 biomarkers yielded best and significant improvements in the C-index. Advancing laboratory techniques allowing simultaneous measurement of many biomarkers are becoming more and more realistic in clinical practice. Whether the biomarkers identified are either involved in the causal pathway contributing to CKD progression, or are markers of its risk, or are merely the end-product of existing pathological processes, remains an important and unresolved question that requires further exploration. A future study on etiology to examine the causal relationship between these biomarkers as risk factors of renal disease would be appropriate, and issues of confounding could then be addressed. Testing for confounding was beyond the scope of this prediction study; however, we were able to investigate the importance of each of the predictors in the optimal model. Baseline UACR was found to have the largest impact on eGFR decline, and only the omission of baseline UACR or systolic blood pressure allowed inclusions of other novel biomarkers into the model. The combination of multiple biomarkers in the final, optimal model appears to be more accurate in risk stratification for accelerated renal function decline in patients with type 2 diabetes.

There are some studies in literature that use a multi-biomarker approach for risk prediction in CKD. A recent study showed that the combination of a panel of biomarkers including inflammation, fibrosis, and cardiac stretch and injury improved prediction of death in a Canadian CKD cohort; however, this study was conducted in a cohort with different CKD etiology [32]. Additionally, in another study of multiple protein biomarkers, 17 urinary and 7 plasma biomarkers were evaluated to predict progression. C-terminal FGF-23 and VEGF-A were found to be associated with the end point independent of urine albumin/creatinine. In that study many biomarkers were tested one by one, but did not use a combined biomarker approach to predict renal disease progression [33]. Furthermore, a panel of multiple urinary cytokines was found to predict rapid renal function decline in overt diabetic nephropathy [34]. However, that study included a heterogeneous population of patients with both type 1 and type 2 diabetes. Finally, in a *post-hoc* study from the IRMA-2 trial showed that multiple biomarkers of endothelial dysfunction and possibly inflammation were predictors of progression to diabetic nephropathy in patients with type 2 diabetes and microalbuminuria independent of traditional risk markers [35].

Advances in high throughput analytical methods has fueled novel biomarker discovery. Two such platforms, namely proteomics and metabolomics, have shown promise in multi-biomarker discovery for the diabetic CKD. A urinary peptide classifier, consisting of 273 defined urinary peptides, was recently discovered as a good classifier in patients with CKD [36] and validated in an independent cohort as a predictor of albuminuria progression in patients with type 2 diabetes [37]. Furthermore, a panel of 13 metabolites linked with mitochondrial metabolism was significantly reduced in CKD patients with diabetes compared to healthy controls [38], and the combination of plasma metabolites butenoylcarnitine and histidine, and urine metabolites glutamine, tyrosine, and hexoses were able to predict the progression from micro- to macroalbuminuria in patients with type 2 diabetes [39].

Interestingly in our study, HbA_1c_ and duration of diabetes were not strong predictors of eGFR decline, whereas albuminuria was identified as the strongest predictor. The exclusion of HbA_1c_ and duration of diabetes from the reference model may be due to small variations in these parameters within this population. Regarding albuminuria, there is evidence that demonstrates albuminuria as a strong risk predictor of renal function loss in patients with type 2 diabetes [40–43]. Moreover, experimental data show that increased albumin exposure to the tubuli causes tubulo-interstitial damage through activation of pro-inflammatory mediators, which leads to a progressive decline in glomerular and tubular function, ultimately culminating in end-stage renal disease [44, 45]. Our data on albuminuria as a strong predictor of eGFR decline are in line with this and highlight the importance of screening for high albuminuria to identify individuals at risk of progressive renal function loss. At the same time, it may be interesting to explore the predictive ability of urine biomarkers alongside albuminuria for renal disease progression as urine is considered quite a suitable substrate to measure biomarkers linked to kidney disease due to the practical advantages of collecting urine compared to blood samples. Since our study measured biomarkers in blood, we are unable to speculate if urine biomarkers, or the combination of both blood and urine markers, would yield similar predictive capabilities.

There are strengths and limitations to this study. A clear strength is the use of a multi-marker, multi-pathway approach for identifying and testing biomarkers in a population of patients with type 2 diabetes over approximately 4 years of follow-up. The clear limitation is the measurement of multiple biomarkers in a small sample size. However, as advancing laboratory techniques generate larger amounts of data, methods of data analysis to accommodate “big data” with smaller sample sizes are needed. The rigorous statistical method of the LASSO regression allowed for modeling many biomarkers in the small sample size, and multiple imputation was used to avoid truncating observations due to missing data. The true predictive capacity of the model could have been overestimated due to the prediction model being developed and tested in the same sample, and we do agree that external validation is necessary. In the absence of external validation, we performed internal bootstrap validation in an attempt to minimize this limitation [46]. GFR was estimated using a serum creatinine-based equation instead of by direct measurement, which may have contributed to misclassification bias. However, this could have only resulted in an underestimation of the strength of the reported associations. We chose to omit five biomarkers from our analysis due to many missing or below LOD values. While the exclusion of these biomarkers from our analysis may have resulted in an underrepresentation of pathways, the omission of biomarkers could have only underestimated the predictive ability of the biomarker panel. Additional limitations include the lack of information concerning insulin use, diet, and renin-angiotensin-aldosterone system medication type and dose, which clearly represent unmeasured confounders in our study.

In conclusion, novel biomarkers may provide deeper understanding into the pathophysiology of CKD or diabetic nephropathy but identification of progression-associated molecular pathways via biomarkers as proxy may also help to identify novel therapeutic targets. We identified a novel panel of biomarkers representing different pathways of renal damage, including inflammation, fibrosis, angiogenesis, and endothelial function. This combined panel improved prediction of accelerated renal function decline in patients with type 2 diabetes on top of established risk markers. The results of this study need to be validated in a large, prospective cohort to validate and assess its applicability in a broad type 2 diabetes population.

## Supporting Information

S1 Appendix Selection of biomarkers. (DOC)

S2 Appendix Biomarker assay information. (DOC)

S3 Appendix Extended statistical methods. (DOC)

S1 Fig LASSO selection of established risk markers. (DOC)

S1 Table Impact of omitting variables from the model. (DOC)
