# Supplementary material for: A Panel of Novel Biomarkers Representing Different Disease Pathways Improves Prediction of Renal Function Decline in Type 2 Diabetes
Source: PLoS One. 2015 May 14;10(5):e0120995. doi: 10.1371/journal.pone.0120995 (PMC4431870; doi:10.1371/journal.pone.0120995)
Supplement: S1 Appendix — (DOC) [file pone.0120995.s001.doc]

**Supplementary Appendix 1.** Selection of biomarkers

Biomarker candidates were selected following three strategies in the realm of integrative evaluation, namely a manual consolidation effort and two algorithmic approaches. Common denominator of the procedures was evaluation of a candidate’s relevance in the context of molecular processes and pathways of putative relevance in diabetic nephropathy. Respective molecular pathways were extracted from the title and abstracts of publications retrieved with the following NCBI PubMed query: ("diabetic nephropathy"[ti] OR "diabetic nephropathies"[ti]) AND (pathway[ti] OR pathways[ti]).

Manual consolidation of biomarker candidates started with a literature review resting on PubMed MeSH annotation “biological markers” [mh] and “Diabetic Nephropathies” [mh]. Utilizing mechanistic context literature reviewing based on NCBI GeneRIF (ftp://ftp.ncbi.nih.gov/gene/GeneRIF/) and tissue specific expression information retrieved from a gene expression dataset on 32 human tissues (http://www.ncbi.nlm.nih.gov/geo/query/acc.cgi?acc=GSE7905) as well as from abundance information available in GeneCards (http://www.genecards.org) twelve biomarker candidates were selected for experimental evaluation utilizing ELISA-based assays: zinc-binding alpha-2-glycoprotein 1 (AZGP1); endostatin (fragment of COL18A1); connective tissue growth factor (CTGF); fibroblast growth factor 23 (FGF23); galectin-3 (LGALS3); monocyte chemoattractant protein-1 (CCL2); nephrin (NPHS1); podocin (NPHS2); neuropilin-1 (NRP1); amino terminal pro C-type natriuretic peptide (NT-proCNP); sclerostin (SOST), and tumor necrosis factor receptor 2 (TNFR2).

The second biomarker selection procedure utilized a molecular model representation of diabetic nephropathy [1]. In brief, the methodology consolidates Omics signatures associated with diabetic nephropathy on a molecular interaction network, and segments the induced subgraph into molecular units utilizing topological criteria. Based on this approach the following biomarker candidates were selected for multiplexed (Luminex-based) analysis: chitinase 3-like 1 (YKL-40); chemokine (C-X-C motif) 1 (CXCL1); chemokine (C-X-C motif) 10 (CXCL10); epidermal growth factor (EGF); growth hormone 1 (GH1); hepatocyte growth factor (HGF); interleukin 1 beta (IL1B); leptin (LEP); a set of matrix metallopeptidases (MMP13, MMP2, MMP7, MMP8); tyrosine kinase (TEK); tumor necrosis factor receptor superfamily, member 1A (TNFR1), and vascular endothelial growth factor A (VEGF-A). This selection procedure also included CCL2 already being derived as candidate from manual consolidation.

The third biomarker selection procedure rested on a ranking procedure of consolidated Omics signatures utilizing annotation from Genecards [2]. Next to the candidates CXCL1, CXCL10, EGF, IL1B and MMP2 also selected based on the molecular model analysis, CCL2 selected on the basis of the molecular model as well as on the basis of manual consolidation, and endostatin also selected based on manual evaluation, Interleukin 1 alpha (IL1A) and matrix metallopeptidases 1 (MMP1) were included as additional candidates for experimental evaluation.

**References**

1. Heinzel A, Muhlberger I, Fechete R, Mayer B, Perco P. Functional molecular units for guiding biomarker panel design. Methods Mol Biol 2014;1159:109-133
2. Stelzer G, Dalah I, Stein TI, Satanower Y, Rosen N, Nativ N, Oz-Levi D, Olender T, Belinky F, Bahir I, Krug H, Perco P, Mayer B, Kolker E, Safran M, Lancet D. In-silico human genomics with GeneCards. Hum Genomics 2011 Oct;5(6):709-717
