# Supplementary material for: A Panel of Novel Biomarkers Representing Different Disease Pathways Improves Prediction of Renal Function Decline in Type 2 Diabetes
Source: PLoS One. 2015 May 14;10(5):e0120995. doi: 10.1371/journal.pone.0120995 (PMC4431870; doi:10.1371/journal.pone.0120995)
Supplement: S2 Appendix — (DOC) [file pone.0120995.s002.doc]

**Supplementary Appendix 2.** Biomarker assay information

At the time of the study, most assays were available directly from the manufacturer, except for FGF23 and Endostatin (Biomedica/Biomarker Design Forschungs GmbH (BDF), Vienna, Austria) which were under development and validation according to the International Conference of Harmonization (ICH). Both assays met all ICH standards.

Regarding the limit of detection, for Biomedica/BDF assays, the Lower Limit of Detection (LLD) was defined as the calculated concentration of the lowest standard point plus three times the standard deviation. For Fibrogen (FibroGen Inc., San Francisco, USA), the detection limit of the CTGF assay was 4 pmol/l, and intra- and interassay variations were 6 and 20%, respectively. For R&D Systems (R&D Systems, Minneapolis, USA) assays, the Minimum Discriminatory Difference (MDD) was determined by adding two standard deviations to the mean optical density value of twenty zero standard replicates and calculating the corresponding concentration. For USCN (USCN Life Science Inc., Wuhan, Hubei, China) assays, the LLD was defined as the lowest protein concentration that could be differentiated from zero. It was determined by adding two standard deviations to the mean optical density value of twenty zero standard replicates and calculating the corresponding concentration.

Regarding stability of the biomarkers, we have no information about long term stability. USCN recommends to avoid repeated freeze/thaw cycles. R&D Systems and Biomedica/BDF have tested the freeze/thaw stability and state that the analytes are stable for at least 3 freeze/thaw cycles.

| **Biomarker** | **Assay** | **Manufacturer** |
| --- | --- | --- |
| Endostatin (Frag.COL18A1) | ELISA | Biomedica/BDF |
| Amino terminal pro C-type natriuretic peptide (NT-proCNP) | ELISA | Biomedica/BDF |
| Fibroblast growth factor 23 (FGF23) | ELISA | Biomedica/BDF |
| Sclerostin (SOST) | ELISA | Biomedica/BDF |
| Connective tissue growth factor (CTGF) | ELISA | Fibrogen |
| Podocin (NPHS2) | ELISA | USCN |
| Zinc-binding alpha-2-glycoprotein 1 (AZGP1) | ELISA | USCN |
| Nephrin (NPHS1) | ELISA | USCN |
| Neuropilin-1 (NRP1) | ELISA | USCN |
| Tumor necrosis factor receptor-2 (TNFR2) | ELISA | R&D Systems |
| Monocyte chemoattractant protein-1 (CCL2) | Multiplex | R&D Systems |
| Tumor necrosis factor receptor-1 (TNFR1) | Multiplex | R&D Systems |
| Chitinase 3-like 1 (YKL-40) (ng/mL) | Multiplex | R&D Systems |
| Chemokine (C-X-C motif) 1 (CXCL1) | Multiplex | R&D Systems |
| Chemokine (C-X-C motif) 10 (CXCL10) | Multiplex | R&D Systems |
| Matrix metallopeptidase 1 (MMP1) | Multiplex | R&D Systems |
| Matrix metallopeptidase 2 (MMP2) | Multiplex | R&D Systems |
| Matrix metallopeptidase 7 (MMP7) | Multiplex | R&D Systems |
| Matrix metallopeptidase 8 (MMP8) | Multiplex | R&D Systems |
| Matrix metallopeptidase 13 (MMP13) | Multiplex | R&D Systems |
| Leptin (LEP) | Multiplex | R&D Systems |
| Tyrosine kinase (TEK) | Multiplex | R&D Systems |
| Vascular endothelial growth factor-A (VEGF-A) | Multiplex | R&D Systems |
| Hepatocyte growth factor (HGF) | Multiplex | R&D Systems |
| Growth hormone 1 (GH1) | Multiplex | R&D Systems |
| Interleukin-1 alpha (IL1A) | Multiplex | R&D Systems |
| Interleukin-1 beta (IL1B) | Multiplex | R&D Systems |
| Epidermal growth factor (EGF) | Multiplex | R&D Systems |
