# Supplementary material for: A Panel of Novel Biomarkers Representing Different Disease Pathways Improves Prediction of Renal Function Decline in Type 2 Diabetes
Source: PLoS One. 2015 May 14;10(5):e0120995. doi: 10.1371/journal.pone.0120995 (PMC4431870; doi:10.1371/journal.pone.0120995)
Supplement: S3 Appendix — (DOC) [file pone.0120995.s003.doc]

**Supplementary Appendix 3.** Extended statistical methods

*a. Pooling of results across the 20 imputed data sets*

For univariate or single-biomarker models, we applied Rubin’s rules to pool results across the 20 imputed data sets [1].

To avoid the LASSO to select different variables in each of the imputed data set, we estimated regression coefficients after pooling the imputed data sets into one ‘stacked’ data set. Since the LASSO does not involve the estimation of standard errors of regression coefficients, this is a valid approach to estimate the posterior modes of the regression coefficients, since in standard linear regression analyses posterior modes obtained by proper pooling and by a ‘stacked data’ analysis are approximately similar.

However, when the tuning parameter which controls the amount of restriction in the LASSO is optimized by cross-validation, the dependency of multiple data lines contributed by a single patient must be accounted for. Thus, our implementation of cross-validation involved leaving out all 20 imputed data lines of a patient simultaneously, and repeating this for each patient in turn. We validated this approach by comparing the such-optimized tuning parameter with the distribution of optimized tuning parameters obtained when each imputed data is analyzed separately. In addition, we compared the final number of selected variables in the pooled analysis with the distribution of selected variables across the 20 individual analyses (data not shown). We became aware of the existence of more sophisticated ways of applying the LASSO to multiply imputed data only after completing data analysis of this project [2].

*b. Computation of predicted probabilities of accelerated eGFR function decline*

From a predicted eGFR slope (*Y*) and its standard error for individual prediction (*S*), we derived the probability of accelerated eGFR decline (*P*), defined as a eGFR slope < -3 mL/min/1.73m^2^/year, as follows, making use of a normal approximation: *P*=({-3-*Y*}/*S*), where (*z*) denotes the cumulative distribution function of the standard normal distribution, evaluated at *z*. *S* was defined as the bootstrap estimate of the root mean squared error of prediction. The probabilities *P* are shown and compared between patients with accelerated and non-accelerated eGFR decline in Figure 2.

**References**

1. White IR, Royston P, Wood AM. Multiple imputation using chained equations: Issues and guidance for practice. Stat Med 2011 Feb 20;30(4):377-399
2. Chen Q, Wang S. Variable selection for multiply-imputed data with application to dioxin exposure study. Stat Med 2013 Sep 20;32(21):3646-3659
