# Supplementary material for: A Panel of Novel Biomarkers Representing Different Disease Pathways Improves Prediction of Renal Function Decline in Type 2 Diabetes
Source: PLoS One. 2015 May 14;10(5):e0120995. doi: 10.1371/journal.pone.0120995 (PMC4431870; doi:10.1371/journal.pone.0120995)
Supplement: S1 Fig — (DOC) [file pone.0120995.s004.doc]

**Supplemental Figure 1.** LASSO selection of established risk markers: cross validated mean squared error (Y-axis; red bullets; MSE) vs. amount of restriction (X-axis; log(Lambda)). Vertical bars refer to standard errors across the 82 cross-validations. Predictive accuracy reached a plateau after step 21 (21^st^ bullet from the right). At this step, the following established risk markers were selected: baseline UACR, current vs. never smoker, sex, systolic and diastolic blood pressure, use of oral diabetic medication, and baseline eGFR.
