# Supplementary material for: A Panel of Novel Biomarkers Representing Different Disease Pathways Improves Prediction of Renal Function Decline in Type 2 Diabetes
Source: PLoS One. 2015 May 14;10(5):e0120995. doi: 10.1371/journal.pone.0120995 (PMC4431870; doi:10.1371/journal.pone.0120995)
Supplement: S1 Table — (DOC) [file pone.0120995.s005.doc]

**Supplemental Table 1.** Impact of omitting variables from the model. Column variables are the newly included variable to the model with their standardized betas if the variable in the row was omitted from the full model. Standardized betas represent percent change in eGFR per standard deviation increment in biomarker level. Eliminating the variables baseline eGFR, the use of oral diabetic medication, NT-proCNP or CCL2 did not include any new predictors to the model. Only the omission of baseline UACR or systolic blood pressure resulted in relevant inclusions of other novel biomarkers into the model (defined as inclusions of predictors with a standardized coefficient of >5%).

|  | DBP | VEGF-A | GH1 | LEP | HGF | CXCL1 | CXCL10 | TNFR2 | AZGP1 | FGF23 | Frag.COL18A1 |
| --- | --- | --- | --- | --- | --- | --- | --- | --- | --- | --- | --- |
| Baseline UACR | 0.06 | 7.3 | 1.1 | 5.9 | 5.3 | 17.6 | 5.2 | 0.0 | 18.6 | 9.7 | 0.0 |
| Baseline eGFR | 0.0 | 0.0 | 0.0 | 0.0 | 0.0 | 0.0 | 0.0 | 0.0 | 0.0 | 0.0 | 0.0 |
| CTGF | 0.0 | 0.0 | 0.5 | 0.0 | 0.0 | 0.0 | 0.0 | 0.0 | 0.0 | 0.0 | 0.0 |
| TEK | 0.0 | 0.0 | 1.0 | 0.0 | 2.0 | 0.0 | 0.0 | 0.0 | 0.0 | 0.0 | 0.0 |
| Systolic blood pressure | 0.8 | 3.1 | 0.8 | 0.0 | 7.1 | 1.5 | 0.3 | 0.0 | 8.7 | 0.0 | 0.0 |
| MMP2 | 0.0 | 0.0 | 0.0 | 0.0 | 0.0 | 0.0 | 0.0 | 0.0 | 0.0 | 2.5 | 0.0 |
| MMP7 | 0.0 | 0.0 | 0.0 | 0.0 | 1.0 | 0.0 | 0.0 | 0.0 | 0.0 | 0.0 | 0.0 |
| Current vs. never smoker | 0.0 | 0.0 | 0.0 | 4.2 | 0.2 | 0.0 | 2.5 | 0.0 | 0.0 | 0.0 | 0.0 |
| TNFR1 | 0.0 | 0.0 | 0.8 | 0.0 | 1.1 | 0.0 | 0.0 | 0.0 | 0.0 | 0.0 | 0.0 |
| MMP1 | 0.0 | 0.0 | 0.0 | 0.0 | 0.8 | 0.0 | 0.0 | 0.0 | 0.0 | 0.0 | 0.0 |
| MMP8 | 0.0 | 0.0 | 0.4 | 1.4 | 0 | 0.0 | 0.2 | 0.0 | 0.0 | 0.0 | 0.0 |
| NPHS2 | 0.0 | 0.0 | 0.0 | 0.1 | 0.5 | 0.0 | 0.0 | 0.0 | 0.0 | 0.0 | 0.0 |
| Oral diabetic medication | 0.0 | 0.0 | 0.0 | 0.0 | 0.0 | 0.0 | 0.0 | 0.0 | 0.0 | 0.0 | 0.0 |
| Sex | 0.0 | 0.0 | 0.0 | 3.0 | 0.5 | 0.0 | 0.0 | 0.0 | 0.0 | 0.0 | 0.0 |
| SOST | 0.0 | 0.0 | 0.0 | 1.4 | 0.3 | 0.0 | 0.0 | 0.0 | 0.0 | 0.0 | 0.0 |
| YLK-40 | 0.0 | 0.0 | 0.0 | 0.0 | 1.0 | 0.0 | 0.0 | 0.0 | 0.0 | 0.0 | 0.0 |
| NT-proCNP | 0.0 | 0.0 | 0.0 | 0.0 | 0.0 | 0.0 | 0.0 | 0.0 | 0.0 | 0.0 | 0.0 |
| CCL2 | 0.0 | 0.0 | 0.0 | 0.0 | 0.0 | 0.0 | 0.0 | 0.0 | 0.0 | 0.0 | 0.0 |
| MMP13 | 0.0 | 0.0 | 0.0 | 0.2 | 0.3 | 0.0 | 0.0 | 0.0 | 0.0 | 0.0 | 0.0 |
